# Barriers to cardiovascular magnetic resonance imaging scan performance and reporting by cardiologists: a systematic literature review

**DOI:** 10.1093/ehjimp/qyaf010

**Published:** 2025-01-22

**Authors:** Tesfamariam Betemariam, Abeba Aleka, Ekram Ahmed, Tinsae Worku, Yonas Mebrahtu, Emmanuel Androulakis, Steffen E Petersen, Rocco Friebel

**Affiliations:** William Harvey Research Institute, NIHR Barts Biomedical Research Centre, Queen Mary University London, Charterhouse Square, London EC1M 6BQ, UK; Barts Heart Centre, St Bartholomew's Hospital, Barts Health NHS Trust, West Smithfield, London EC1A 7BE, UK; Department of Health Management, University of Global Health Equity, Kigali, Rwanda; Department of Public Health and Health Equity, Vrije Universite, Amsterdam, The Netherlands; Department of Health Management, University of Global Health Equity, Kigali, Rwanda; Department of Internal Medicine, Ascension Saint Joseph Hospital, Chicago, IL, USA; Cardiovascular Imaging Department, Royal Brompton and Harefield Hospital NHS Foundation Trust, London, UK; William Harvey Research Institute, NIHR Barts Biomedical Research Centre, Queen Mary University London, Charterhouse Square, London EC1M 6BQ, UK; Barts Heart Centre, St Bartholomew's Hospital, Barts Health NHS Trust, West Smithfield, London EC1A 7BE, UK; Department of Health Policy, London School of Economics, London, UK

**Keywords:** CMR, challenges, cardiologists

## Abstract

**Aims:**

Cardiovascular magnetic resonance (CMR) imaging plays a pivotal role in diagnosing and managing cardiovascular diseases. Its use has shown sustained growth over the past years. However, there is considerable variability in the use and reporting of CMR scans worldwide. This review provides synthesis of evidence on the barriers and challenges to performing CMR scans by cardiologists and gain insights into the variations in CMR scan practices across different countries.

**Methods and results:**

We systematically reviewed the literature from 1 January 2003 up to 13 November 2023. We searched four databases (Ovid Medline, Embase, Web of Science, and Scopus) and hand-searched the references in the included articles, complemented by expert feedback. Articles were double screened against pre-defined inclusion and exclusion criteria. We conducted risk of bias using the JBI critical appraisal tool, and we analysed information using a narrative synthesis of results. We identified 14 857 articles, with 13 articles meeting the inclusion criteria. The key barriers were the limited availability of CMR scanners, resulting in extended waiting times, the high service cost, and limited training opportunities and the lack of a structured curriculum. The main practice variations identified were geographical disparities in CMR use. Worldwide, the majority of CMR training programmes are situated in radiology departments.

**Conclusion:**

Barriers to CMR use by cardiologists range from access to scanners and prohibitive costs to disparities in familiarity with CMR technology. Geographic variations and heterogeneity in training programmes underscore the influence of systemic factors such as healthcare infrastructure, reimbursement policies, and unstandardized training curricula.

## Graphical Abstract

**Graphical Abstract:**
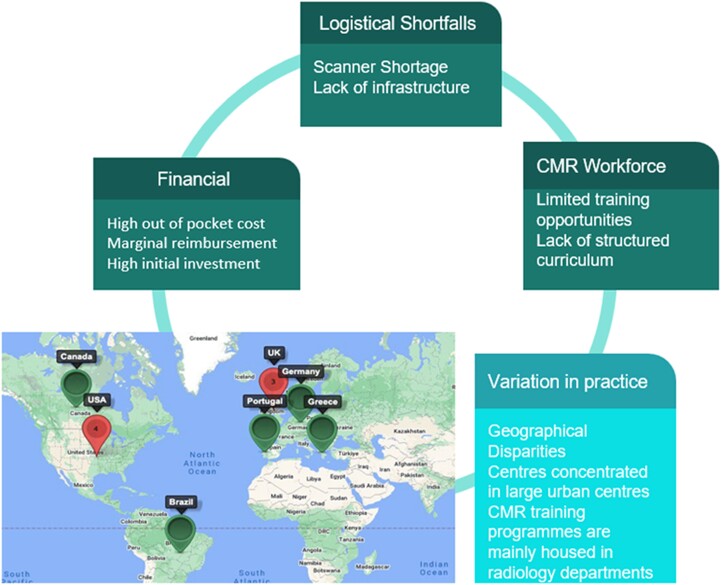
This figure highlights the challenges in global implementation of cardiovascular magnetic resonance, including logistical shortfalls, financial barriers, and workforce limitations. It also illustrates geographical disparities.

## Introduction

Cardiovascular magnetic resonance (CMR) imaging has evolved into a robust diagnostic tool over the last few decades, extensively used in various clinical and research fields.^1–3^ The global utilization of CMR has significantly increased, with Europe alone experiencing a 3.8-fold rise in CMR use between 2011 and 2022.^4^ Similar trends have been observed in the USA and Canada.^5^

CMR is now widely regarded as the gold standard for non-invasive quantitative assessment of cardiac volumes and structures, offering superior accuracy in diagnosing, monitoring, and prognosticating cardiac disorders.^6^ The European Society of Cardiology (ESC) included CMR in all but one of its 27 guidelines in 2023, reflecting a 146% increase since 2015.^7^ Recent advancements in imaging technology, including artificial intelligence (AI), have further expanded its applicability in detecting and treating cardiovascular diseases.^8^ These improvements allow for detailed examination of cardiac anatomy, function, and tissue characterization without exposing patients to ionizing radiation.^9^ A large multicentre study has demonstrated that CMR significantly enhances patient management and is highly beneficial in predicting risks for patients with ischaemic cardiomyopathy (ICM) and coronary artery disease (CAD).

Even with its widespread recognition, CMR remains significantly underutilized globally. Less than 30% of European Observational Research Programme (EORP) cardiomyopathy cohort participants underwent CMR exams despite international clinical guidelines advocating its use.^10^ This underutilization highlights a critical gap in implementing best practices in cardiac care. The disparity in CMR access and use across different countries and regions raises significant equity and quality concerns. In many areas, logistical issues, workforce shortages, and financial constraints impede the adoption of CMR. These challenges often result in prolonged scan waiting times, potentially delaying critical diagnostic information. Additionally, variations in how scans are requested, conducted, and reported further exacerbate the inconsistency in CMR utilization. A significant factor contributing to the limited availability of CMR is the high investment required for its infrastructure, leading to its concentration in a few large, specialized centres. However, emerging evidence suggests that CMR is effective and cost efficient in the long term, particularly for evaluating CAD.^11^

Various types of professionals engage in CMR practice, including cardiologists, radiologists, and nuclear medicine specialists.^12^ Cardiologists play a unique role, starting from referring patients with appropriate indications, accurately interpreting scans, and making treatment decisions.^13^ Yet the comparative involvement of cardiologists as CMR practitioners is limited. Hence, the role of these professionals in CMR is vital for effectively utilizing the imaging modality, reducing unnecessary costs, and improving outcomes for cardiac patients. In this systematic review, we synthesize the existing evidence on the challenges and barriers cardiologists encounter in performing and reporting CMR and the global practice variations. The study’s outcomes will support the development of strategies to optimize the utilization of CMR and guide policy and practice to improve existing quality gaps.

## Methods

We conducted a systematic literature review to identify the barriers to the performance of CMR by cardiologists and variations in practice. The study was conducted in line with the Preferred Reporting Items for Systematic Reviews and Meta-Analyses (PRISMA) guidelines.^14^ The study protocol was pre-registered with The International Prospective Register of Systematic Reviews (PROSPERO; registry number: CRD42024513325).

A comprehensive search strategy was developed to guide the review in collaboration with an expert librarian. The approach was created using a combination of keyword and subject heading searches related to CMR. We utilized the fundamental concepts of ‘cardiovascular magnetic resonance imaging’ and ‘barriers’. We systematically searched four bibliometric databases (Ovid Medline, Ovid Embase, Web of Science, and Scopus) for relevant studies from 1 January 2003 up to 13 November 2023. We hand-searched the references of all included studies to identify additional articles.

We utilized EndNote version 21 for de-duplication. Titles and abstracts were screened for relevance by four independent double-blinded reviewers (A.K., E.A., T.W., and Y.M.). The full texts of all potentially relevant studies were retrieved and assessed for eligibility against the inclusion and exclusion criteria. We included primary studies focusing on cardiologists, including those in training (fellows, residents), and all articles published after 2003, regardless of geographical context and language. Systematic reviews, conference abstracts, and articles exclusively discussing barriers to performance and reporting by non-cardiology professionals were excluded. Any disagreement between reviewers was resolved by discussion and the judgement of a third reviewer when consensus was not reached. The screening used Rayyan, a machine learning platform, to facilitate systematic reviews.^15^

A pretested data collection template was used for double-blinded data extraction. Descriptive information about each study, including article metadata, study design, sample size, population included, challenges, and practice variations, was recorded.

We conducted the quality assessment using the JBI critical appraisal tools.^16^ Due to the paucity of literature in the subject area, we did not exclude any studies based on the quality of the papers. Lastly, findings were synthesized using a narrative approach.

## Results

**Figure 1:**
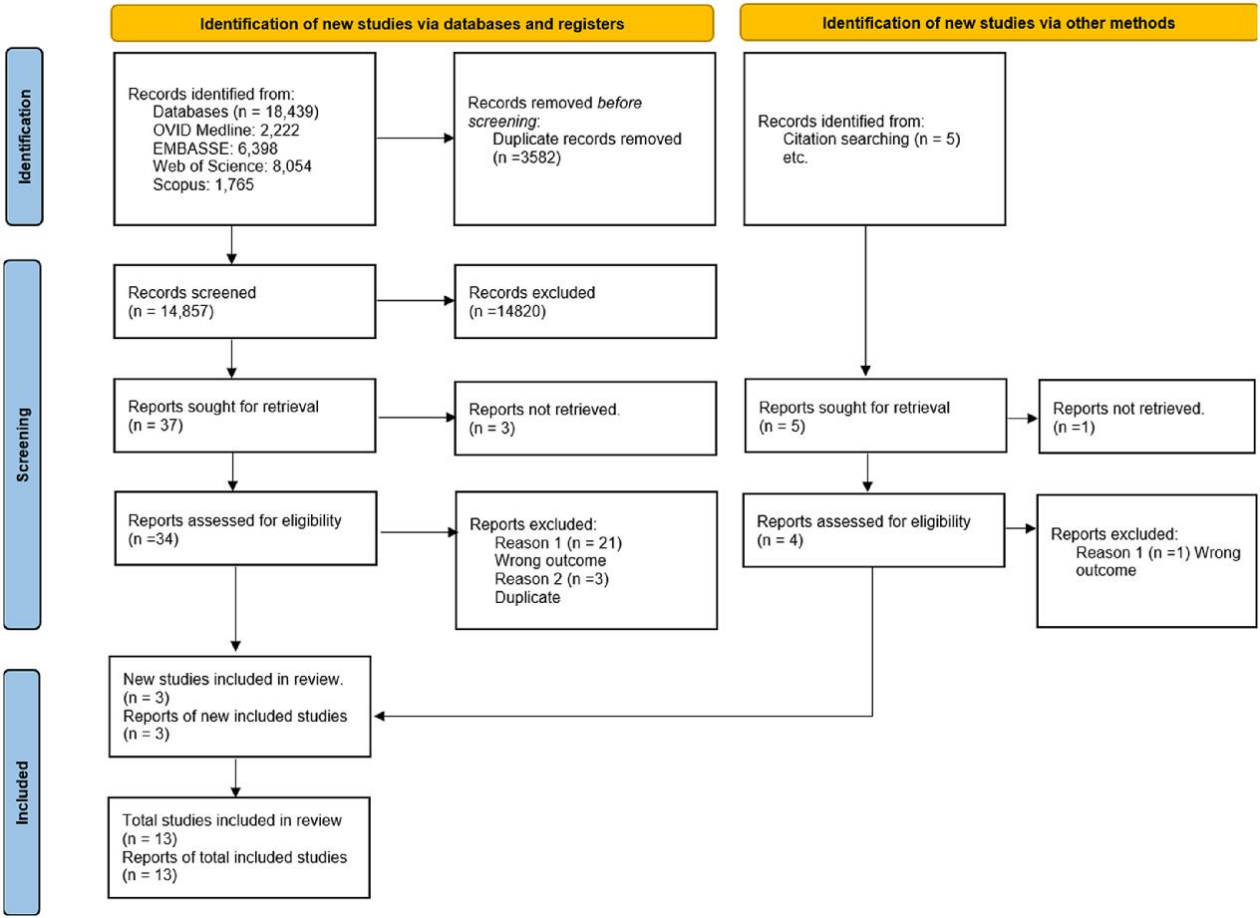
PRISMA flowchart.

Our initial search yielded 18 439 studies. Following de-duplication, 14 857 article titles and abstracts were screened. Thirty-seven articles underwent full-text review, with 14 articles meeting the inclusion criteria (*Figure 1*).

### Characteristics of included studies

Eleven of the studies included were cross-sectional surveys, and two were reports by professional societies. Across all studies, each of the following journals published two articles: the *Journal of Cardiovascular Magnetic Resonance*, the *Journal of the American College of Cardiology*, and *Radiology Cardiothoracic Imaging*. All studies were published between 2004 and 2023, and >60% (*n* = 8) were published since 2013. Most of the included studies were from North America (USA and Canada) (*n* = 5) and Europe, including the UK (*n* = 5). A small proportion were multi-country (*n* = 2). Brazil was the only low- and middle-income country identified in the review. Among the included papers, only two studies discussed the challenges specific to paediatric cardiac imaging. Lastly, all papers except one were written in English.

### Challenges of CMR performance and reporting

#### Availability, resources, and provider payment

The majority of articles reported limited availability as a primary challenge of CMR utilization. Antony *et al.*^17^ reported that in a survey that covered 281 centres, over two-thirds of surveyed hospitals, ∼78% (221) in the UK, lacked CMR services. These centres referred their patients to other centres for CMR services. Similarly, Portuguese doctors (45%) attributed the low CMR performance rate to a lack of availability, especially in public hospitals.^18^ Additionally, CMR services were available only at medium to large hospitals. Authors described the long waiting time for a CMR due to the lack of widespread availability, being a deterrent for professionals to request scans.^19–21^

Four of the reviewed studies show that high costs are a critical barrier.^18,21–23^ Ferreira *et al.* reported that 36% of their study participants did not refer their patients due to the perception that CMR is more expensive and rated CMR pricier than other imaging modalities surveyed in the study. A global survey by Sierra-Galan *et al.*^22^ reported that high-volume centres were more likely to consider cost a significant obstacle than low-volume centres. The study further demonstrated both low- and high-income countries perceived costs similarly.

The resource-intensive nature of acquiring and maintaining scanners was a significant challenge reported by the studies.^24^ Lack of resources, long scanning protocol duration, and complex technical requirements compared to other imaging technologies are cited as reasons for low CMR utilization.^19,22^

A study by Petersen *et al.*^25^ infers that reimbursement mechanisms significantly drive higher absolute CMR activity in England vs. the USA. Overall, non-invasive cardiac imaging (NICI) activity was higher in the USA due to higher physician fee-for-service compensation and lower reimbursement for CMR services. The difference in Medicare reimbursement rates varies based on whether the imaging is performed in hospital inpatient, outpatient, or physician offices/imaging centres. Additionally, the marginal reimbursement rates for cardiology office-based CMR and the lack of coverage by national health services further constrained its utilization.^26^ Sierra-Galan *et al.* note that high-volume centres disproportionately identify low reimbursement rates as a significant barrier to CMR growth. Similarly, in Canada, more than half of the surveyed sites (53%) considered inadequate reimbursement per scan to be a barrier to further clinical utilization of CMR.^19^ In Portugal, CMR availability is limited in hospitals and not supported by the National Health Service, posing a barrier to its broader clinical adoption.^18^ In the UK, only 38% of centres have dedicated funding for CMR, mainly from primary care trusts.^17^

#### CMR workforce

A need for more professionals proficient in CMR reporting was one of the key barriers identified in this review.^21^ More than half (55%) of responders in a global survey lacked formal certification in CMR.^22^ The lack of familiarity was attributed to two main factors: limited training opportunities and a lack of standardized curricula for dedicated fellowships in the field. These challenges were reported in studies from Portugal, the USA, and the UK.^18,24,25^ A study by Sierra-Galan *et al.* showed that 12% of the surveyed participants from low- and middle-income countries and 8% of high-income countries considered formal training a major impediment to the progress of CMR in their respective countries. Compared to other modalities, like nuclear imaging, having a dedicated rotation for CMR was less common. The scope of training also lacked variety and depth, and only a small number of training programmes owned CMR equipment.^22,24,27^ The main factors for limited training opportunities were attributed to a lack of infrastructure dedicated to training, the number of trained experts, including referring physicians, faculty support, underdeveloped curricula, and constraints of integrating new rotations in an already fixed fellowship programme.^17,24^ Antony *et al.*^17^ reported that only half of the national centres had training programmes in the UK. Although 42% of the centres had level three accredited trainers, some were without active trainers. Most trainees were from England, mostly in and around London.^20^ Although the usefulness of CMR was acknowledged, unfamiliarity with the technique was evident in Portugal. Many who felt confident in interpreting CMR images without an accompanying report were unaware of several contraindications, and 15% mistakenly believed that the technique involved ionizing radiation.^18^

#### Practice variations

The main practice variations identified were geographical disparities in CMR use, especially in countries like the UK, where high-volume centres are concentrated around metropolitan cities like London^17,20^—typically serving larger populations in their catchment areas. Similarly, in Portugal, most of the centres were found in Lisbon, and the physicians were concentrated in these sites.^18^

Globally, the majority of CMR training programmes are conducted in radiology departments. In the past, cardiologists have led the provision of CMR services; however, recent trends indicate a shift towards radiologists increasingly providing these services. This was reported by Taylor *et al.* in 2004, where radiology providers primarily managed clinical CMR activity and training. From 2015 to 2017 in Canada, while there was increased cardiologist participation in clinical reading, radiologists still held higher CMR readership status.^19^ UK national centres are proportionally led by cardiologists, radiologists, or both. Radiographers acquire most scans, and radiologists report twice as many scans as cardiologists, to a lesser extent by supervised trainees from each department.^17^ Across a global study, more than half (62%) were academic radiology department-based programmes.^22^

In specific patient populations, such as paediatrics, unique challenges in performing and reporting CMR limit its widespread utilization. This includes the need for sedation and more complex protocols for patients with congenital heart disease.^21^ Another specific population includes patients with cardiac implantable electronic devices (CIEDs), where 91% of Portuguese doctors perceive the presence of a pacemaker or implantable cardioverter defibrillator to be a contraindication for CMR^18^

## Discussion

Our systematic review identified 13 studies assessing the challenges of CMR performance by cardiologists and practice variations published between 2003 and 2023. The key barriers highlighted were the limited availability of CMR scanners, which resulted in extended waiting times, the high service cost, and cardiologists’ limited training opportunities and the lack of a structured curriculum.

Many of our included studies were concentrated in large centres in Northern America and Europe, highlighting a significant shortage of information from other parts of the world. This aligns with previous literature highlighting the severe inequalities in access to CMR in many countries despite a large cardiovascular disease burden.^28^ Disparities in MRI scanner availability mirror a similar pattern; for instance, as per the WHO Global Health Observatory, MRI densities in Europe range from 0 in Georgia to 34 and 132 per million population in Germany and Monaco, respectively.^29^ When available, they are limited to tertiary hospitals in capital cities. The high perceived cost of imaging is identified as a deterrent to utilizing CMR; this aligns with studies that have identified a higher cost of hospitalization in patients who have received CMR scans.^30^ Acquiring and maintaining CMR labs is a resource-intensive endeavour that was recognized as a major challenge. However, the established cost-effectiveness of CMR and its contribution to cardiac outcomes provide a strong argument for further advocacy for investment in CMR.^31^

Shortage of expertise is identified as a critical factor in cardiologists’ limited utilization of CMR. This could be due to the resource-intensive nature of CMR training programmes. The Society for Cardiovascular Resonance (SCMR) and the European Association of Cardiovascular Imaging (EACVI) have set out international recommendations to guide credentialing institutions in adequate levels of CMR training.^32–34^ Institutions would require appropriate facilities with scanners and case mix, supported by well-structured training programmes and a qualified trainer. Many centres around the world struggle to fulfil these necessities. The evolution of digital programmes, such as virtual training, could provide an opportunity to integrate virtual training platforms into in-person programmes.^35^ This can create opportunities to link highly specialized centres with the manpower and expertise facilitating knowledge sharing.^36^ There are also recent initiatives to reduce the reliance on specialist knowledge through the increasing use of AI-based tools for scan interpretation in recent years.^37^

Countries like the USA, the UK, Germany, France, Japan, Australia, and Canada have in-country accreditation standards for CMR facilities, necessitating a multi-disciplinary team to ensure comprehensive care. In the UK, for instance, a physician on the cardiology, nuclear medicine, or radiology speciality register with the appropriate CMR accreditation can serve as a clinical lead for CMR labs. This increases the risk of CMR training failing to align with the core competencies required from cardiology trainees fully. For instance, in a study that assessed cardiac radiology in Europe, the majority of MRI (71%) examinations were performed by radiologists.^4^ Similarly, in the USA, the number of radiologists performing CMR used to be lower but exceeded cardiology professionals between 2012 and 2019 (1% vs. 0.2%).^38^ Legal constraints in several EU countries exacerbate this imbalance by restricting cardiologists from independently conducting or reporting CMR scans. These regulations deter young professionals from pursuing expertise in CMR.^39^ CMR has set out guidelines recommending the joint development and administration of CMR training programmes to address some of the challenges with interprofessional competition and promote synergy.^32^ Establishing joint diagnostic pathways and multi-disciplinary teams can further bridge the radiologist–cardiologist divide, fostering collaboration and improving patient care.^32,40^ Furthermore, strengthening early Level I training in CMR for general cardiologists could encourage the utilization of CMR and inspire new cardiologists to pursue CMR certification.

Despite recommendations in clinical guidelines, large variations in practice exist. Common pathways for patient diagnosis and investigation and a joint team of cardiovascular imagers could facilitate cooperation among professionals and reduce practice variation, improving the quality of health services provided to patients. Professional societies that comprise multiple specialist groups play a key role in facilitating the above guidelines. Moreover, our review has also identified that reimbursement practices impact the use of CMR. The role of provider payment mechanisms in practice variations has been evidenced in the literature widely.

Possible opportunities to address the challenges include remote image processing and reducing the demand for the CMR workforce. Technology-assisted telemedicine practices have been evolving rapidly, particularly after the pandemic, and are demonstrated to provide high-quality CMR services. Recent advances and protocols in MRI technology allow for accelerated image acquisition; portable scanners in remote areas hold immense promise for increasing access to CMR. Additionally, integrations with AI-based algorithms facilitate interpretation and are likely to produce a significant impact.^41^

Patients with CIED may require CMR imaging for various reasons over the device’s lifetime. However, technical limitations, differing perspectives among radiologists and cardiologists, and inconsistencies in the legal recommendation, like the requirement of a cardiologist’s presence during their CMR scan, may hinder its broad application. Training more Level 3 accredited cardiologists can address limitations and ensure broader, safer application of CMR for patients with CIEDs.^40^

### Strengths and limitations

To the best of our knowledge, this is the first systematic review attempting to summarize evidence regarding the challenges faced by cardiologists and practice variations in CMR scanning. We utilized a comprehensive search strategy aimed at capturing a broad literature base. Due to the paucity of publications in the area, we did not exclude studies identified as having poor quality.

## Conclusion

The rising burden of cardiovascular disease requires improved diagnostic tools to provide high-quality services to patients. Accordingly, the potential of CMR’s utility in cardiac medicine is growing. However, CMR is still underutilized in most parts of the world. This is due to the high initial investment required to set up CMR labs and the lack of trained professionals due to paucity of training programmes. There are also significant challenges associated with practice variations despite robust clinical guidelines. These challenges call for improved cardiovascular policymaking to strengthen the delivery of healthcare services. The approach for this would need to be multifaceted with increased investments into CMR facilities, support for training programme improvement, and utilizing telemedicine technology for a wider reach.

## Lead author biography

Dr Tesfamariam Betemariam is an assistant professor of internal medicine at Addis Ababa University with an MSc in cardiovascular research. His research interests include cardiac imaging, cardiovascular magnetic resonance (CMR), and ischaemic heart disease (IHD). Dr Betemariam is committed to advancing the use of cutting-edge imaging technologies in cardiology and improving diagnostic and treatment strategies for cardiovascular diseases.
